# Postpartum Preeclampsia Manifesting as a Transient Ischemic Attack: A Case Report on the Multidisciplinary Management of a High-Risk Patient

**DOI:** 10.7759/cureus.79833

**Published:** 2025-02-28

**Authors:** Neguemadji Ngardig Ngaba, Xegfred Lou T Quidet, Ali Hanif Bhatti, Henry Nabeta, Abel Akanyijuka, Adrija Mehta, Misbahuddin Khaja

**Affiliations:** 1 Internal Medicine, BronxCare Health System, Icahn School of Medicine at Mount Sinai, New York, USA; 2 Medicine, American University of Antigua, Coolidge, ATG

**Keywords:** african descent, hypertensive disorders, neurological dysfunction, postpartum preeclampsia, transient ischemic attack

## Abstract

Transient ischemic attack (TIA) is a brief episode of neurological dysfunction caused by focal brain ischemia, typically lasting less than one hour without acute infarction. Preeclampsia, a multisystem hypertensive disorder occurring in pregnancy, significantly heightens the risk of stroke, particularly during the postpartum period.

This case report details a 34-year-old Sub-Saharan African woman, gravida 4 para 4, who experienced a TIA characterized by right-sided weakness and slurred speech 13 days after delivering a baby by cesarean section. Upon presentation to the emergency department with symptoms suggesting a minor stroke, clinical examination revealed hypertension and neurological deficits. Imaging studies clarified the absence of acute intracranial pathology but indicated hypoperfusion in the right frontal white matter and significant chronic sinusitis. The patient's elevated blood pressure and clinical conditions were consistent with postpartum preeclampsia.

Management included dual antiplatelet therapy and antihypertensives alongside seizure prophylaxis. The patient's neurological symptoms resolved within 24 hours, and she was discharged on supportive medications, with follow-up arrangements established for various specialties.

This case emphasizes the need for careful monitoring of postpartum women for signs of preeclampsia and cerebrovascular events, particularly in high-risk populations. It highlights the interaction between these conditions and the importance of collaborative multidisciplinary care. Further research is warranted to explore the link between chronic sinusitis and postpartum preeclampsia.

## Introduction

Transient ischemic attack (TIA) is characterized as a brief episode of neurological deterioration triggered by local brain or retinal ischemia. The clinical symptoms typically last less than one hour, and there is no evidence of acute infarction [1].

Preeclampsia is a multisystem hypertensive disorder unique to pregnancy, characterized by widespread endothelial dysfunction and immune dysregulation [2]. Approximately 36% of women with pregnancy-associated strokes (PASs) have comorbid preeclampsia [3], and preeclampsia increases stroke risk during the puerperium up to six-fold [4].

Although preeclampsia affects 3%-8% of all pregnancies [4,5], the overall occurrence of PAS remains rare (34.2 per 100,000 deliveries) [6]. Hauspurg and Jeyabalan [7] suggested that the diagnosis of postpartum preeclampsia should be considered in women with new-onset preeclampsia occurring 48 hours to six weeks postpartum, according to the terminology used by experts and existing literature on the subject [8].

Meaningful research [9] estimates that the prevalence of postpartum preeclampsia ranges between 0.3% and 27.5% of all pregnancies in the United States. The antiangiogenic protein, soluble fms-like tyrosine kinase 1 (sFlt1), is elevated in pregnant women [10,11]. In the postpartum period, circulating inflammatory factors are no longer different between women with early preeclampsia and those with normal pregnancy at one to three years postpartum [12].

A prospective study that collected blood samples before cesarean section noted that women who developed new-onset postpartum preeclampsia had significantly higher sFlt1 levels and a higher sFlt1-to-placental growth factor ratio compared with women who remained normotensive postpartum [13].

To our knowledge, there is a paucity of reported cases of postpartum preeclampsia revealed by TIA. Hence, we undertook this research to report a case of a 34-year-old Sub-Saharan African woman with postpartum preeclampsia discovered after an episode of TIA.

## Case presentation

This case report delineates the clinical presentation, diagnostic evaluation, and management of a 34-year-old female, gravida 4 para 4, with a medical history significant for a recent cesarean delivery and three previous uncomplicated vaginal deliveries. The patient experienced an acute neurological event characterized by right-sided weakness and slurred speech 13 days postpartum.

**Figure 1:**
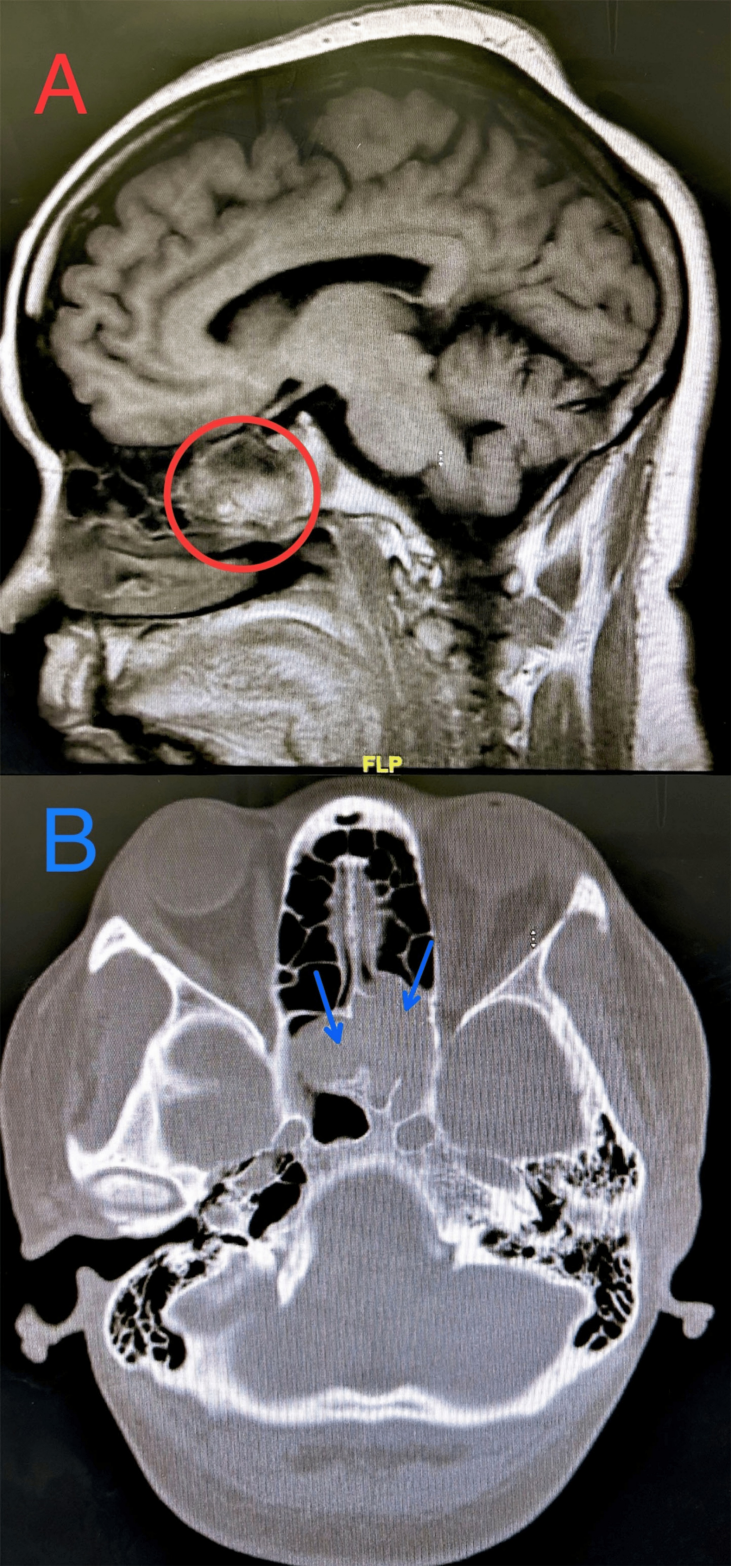
Sphenoid sinus opacification extending to (A) the left orbital apex (red circle) including (B) the left ethmoid sinus (blue arrows)

**Figure 2:**
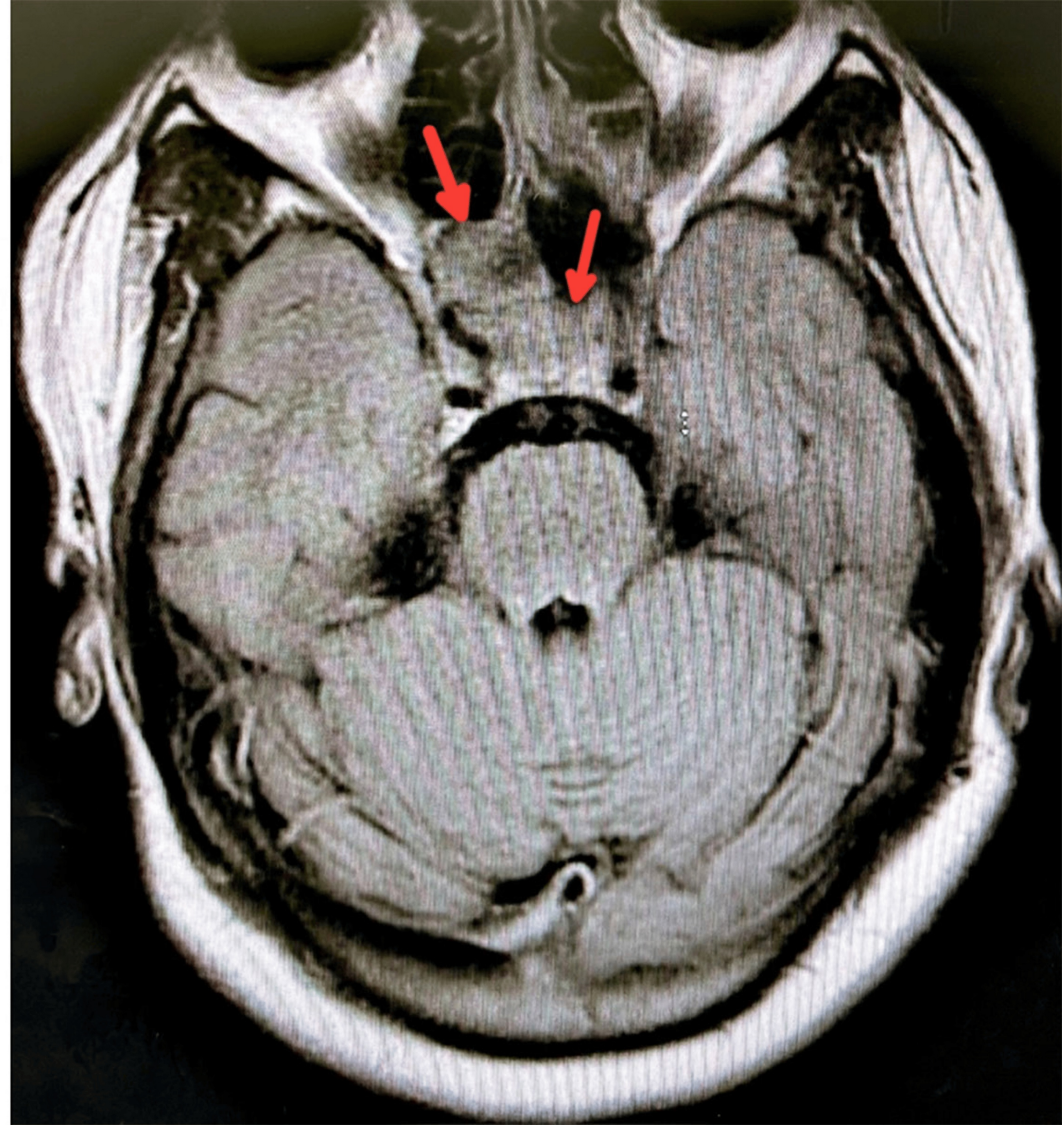
Sphenoid sinus opacification expanding to the left ethmoid sinus (red arrows)

The patient was conveyed by car to the emergency department (ED) 50 minutes following the emergence of sudden right-sided hemiparesis and dysarthria. These symptoms developed after a one-hour car trip, beginning with impaired speech production and evolving into diminished strength in the right upper and lower extremities. The episode lasted about 15 minutes until the initial assessment in the ED. The patient denied preceding trauma, vertiginous episodes, dyspnea, thoracic pain, or cardiac palpitations. In the ED, the patient was conscious, oriented, and exhibited mild expressive aphasia and dysarthria, accompanied by a noticeable drift in the right upper and lower extremities, quantified as a score of 5 on the National Institutes of Health Stroke Scale (NIHSS) [14]. Vital signs were notable for hypertension (156/106 mmHg), tachycardia (104 bpm), and normoxia (99% on ambient air). The patient's physical stature was recorded at 80.5 kg, with a height of 175 cm and a body mass index of 26.3. Ancillary tests, including chest radiography and electrocardiogram, yielded no significant findings. The stroke protocol imaging was initiated with a noncontrast computed tomography (CT) scan of the head, revealing no acute intracranial pathology but significant chronic sinusitis (Figures 1, 2).

CT angiography of the head and neck was unremarkable. CT perfusion imaging indicated no evidence of core infarction yet disclosed hypoperfusion in the right frontal white matter, suggesting a developmental variant in anterior cerebral artery anatomy. The clinical presentation and imaging findings raised suspicions of a TIA or minor stroke. The following medication regimen was prescribed, with specified dosages and durations: dual antiplatelet therapy [15] consisting of aspirin 85 mg daily and clopidogrel 75 mg daily (for 21 days) was initiated, along with labetalol 200 mg every 12 hours [7] for hypertension management, likely related to postpartum preeclampsia. Magnesium sulfate was administered at 2 g per hour for 24 hours as seizure prophylaxis, with recommendations to monitor serum magnesium levels every six hours. Magnesium sulfate was to be discontinued if deep tendon reflexes diminished, if urine output was absent (indicative of oliguria or anuria), or if respiratory depression occurred.

**Figure 3:**
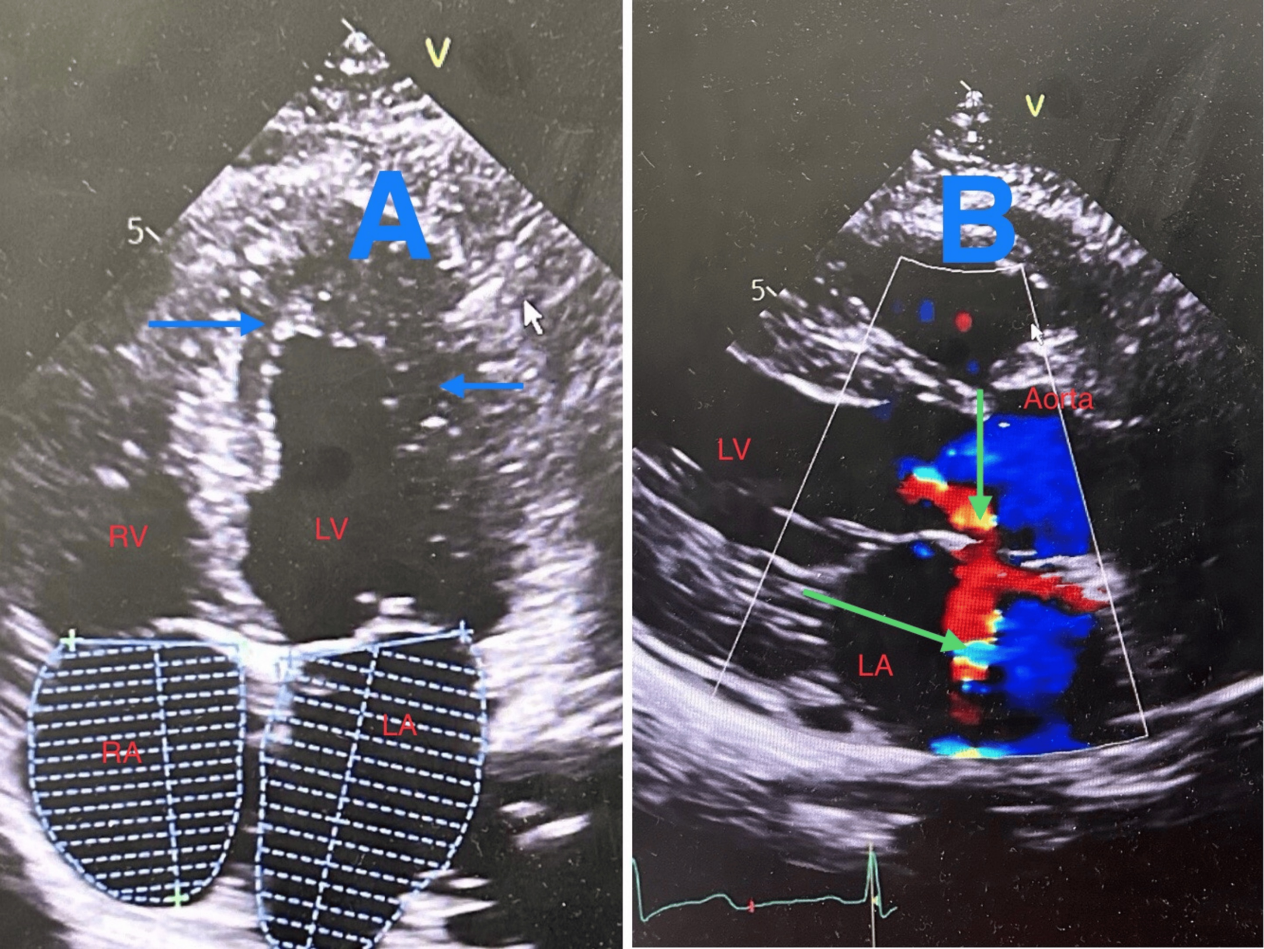
(A) The four-chamber view showing left ventricular wall thickness (blue arrows). (B) Parasternal long axis view showing aortic and mitral valve regurgitation in color Doppler (green arrows) RV: right ventricle; LV: left ventricle; RA: right atrium; LA: left atrium

The patient's neurological deficits were entirely resolved by the first day of hospitalization, reflected by an NIHSS score of 0. An echocardiogram identified left ventricular hypertrophy, diastolic dysfunction, mild regurgitations across multiple valves, and slightly elevated pulmonary artery pressure (Figure 3).

Investigations for systemic lupus erythematosus and antiphospholipid syndrome returned negative. Subsequent brain MRI and magnetic resonance venogram imaging excluded intracranial masses, acute pathologies, venous thrombosis, and posterior reversible encephalopathy syndrome (Table 1).

**Table 1:** Immunologic laboratory test results DRVVT: dilute Russell's viper venom time; ANA: antinuclear antibody; Ig: immunoglobulin; APL: antiphospholipid antibody

Test	Result	Reference range/interpretation
DRVVT screen	32 seconds	≤45 seconds
Cardiolipin antibody IgA	4.3 APL-U/mL	Detected if ≥20.0 APL-U/mL
Cardiolipin antibody IgM	<2.0 APL-U/mL	Detected if ≥20.0 APL-U/mL
Cardiolipin antibody IgG	<2.0 APL-U/mL	Detected if ≥20.0 APL-U/mL
ANA screen	Negative	-
Beta-2 glycoprotein-1 IgM	<2.0 U/mL	Positive if >20.0 U/mL
Beta-2 glycoprotein-1 IgG	<2.0 U/mL	Positive if >20.0 U/mL
Beta-2 glycoprotein-1 IgA	2.2 U/mL	Positive if >20.0 U/mL

The patient's hypertension showed improvement throughout her stay. The follow-up timeline after discharge was as follows: the patient was discharged on the third day with prescriptions for clopidogrel (to complete a total of 21 days), aspirin, and labetalol. Outpatient follow-up appointments were recommended with neurology within one month for TIA management, with a primary care physician within two weeks for routine care, with gynecology within one week for postpartum preeclampsia management, and with otolaryngology within one week to address the incidental finding of chronic sinusitis on imaging.

## Discussion

The patient presented to the emergency room showing stroke-like symptoms 13 days postpartum. The association between stroke-like symptoms and preeclampsia is notably significant, mainly due to the cerebrovascular implications inherent in both conditions. Research by Bushnell and Chireau [16] illustrates the increased risk of cerebrovascular events during and after pregnancy among individuals with preeclampsia, emphasizing the necessity for vigilant monitoring and management of these patients.

Postpartum preeclampsia, albeit relatively uncommon, usually appears within 48 hours of newborn delivery but possibly exhibits up to six weeks postpartum [17]. This interval correlates with our patient's admission 13 days after her newborn delivery, pointing to a possible diagnosis of postpartum preeclampsia.

Further complicating the clinical picture is the observation that being of African American descent is a substantial risk factor for the development of preeclampsia [18,19]. This is particularly relevant given that our patient is of Sub-Saharan African descent.

During the evaluation, the patient displayed elevated blood pressure, which aligns with the diagnostic criteria for postpartum preeclampsia. This condition is defined by two measurements of systolic blood pressure exceeding 140 mmHg and diastolic blood pressure exceeding 90 mmHg, obtained more than four hours apart and without alternative etiologies [7]. Elevated blood pressure is closely linked to increased risks of cerebrovascular complications [20], which necessitates prompt management of new-onset hypertension.

The American College of Obstetricians and Gynecologists (ACOG) recommends using rapidly acting antihypertensives, such as intravenous labetalol, hydralazine, or oral nifedipine, as first-line agents for managing severe hypertension [21]. Consequently, our patient was initiated on labetalol, administered twice daily, stabilizing her blood pressure. Additionally, she received magnesium sulfate at a rate of 2 g/hour for seizure prophylaxis, following ACOG recommendations for postpartum hypertension accompanied by symptoms such as headaches, blurred vision, or severe hypertension.

Interestingly, the brain imaging findings did not correspond to the patient’s clinical symptoms. A pertinent study by Dennis et al. [22] found that among 120 CT scans performed on patients with TIAs, only a tiny percentage (12%) showed a focal area of hypodensity correlated with the patient's clinical presentations. In our case, MRI findings indicated sinusitis, a condition identified as the most prevalent finding in a study by Shobeiri and Torabinejad [23], which reported that 10 out of 42 patients exhibited sinusitis on brain imaging.

## Conclusions

This case underscores the complexity of diagnosing and managing acute neurological events in postpartum patients, highlighting the importance of comprehensive evaluation and multidisciplinary care to ensure favorable management outcomes. Deep research is needed to investigate the relationship between chronic sinusitis and the possibility of developing postpartum preeclampsia.
